# Social fish have larger brains and greater relative telencephalon sizes: support for the social brain hypothesis from wild, intraspecific comparisons

**DOI:** 10.1098/rspb.2025.1169

**Published:** 2025-10-29

**Authors:** Bin Ma, Aneesh P. H. Bose, Boyd Dunster, Boshan Zhu, Etienne Lein, Weiwei Li, Alex Jordan

**Affiliations:** ^1^ Behavioural Evolution Lab, Max Planck Institute of Animal Behavior, Radolfzell, Baden-Württemberg, Germany; ^2^ Department of Biology, University of Konstanz, Konstanz, Baden-Württemberg, Germany; ^3^ Department of Wildlife, Fish & Environmental Studies, Swedish University of Agricultural Sciences (SLU), Umeå, Sweden

**Keywords:** brain size, cichlid, social complexity, social brain hypothesis

## Abstract

The social brain hypothesis (SBH) posits that complex social environments drive the evolution of larger brains and enlargement of specific brain regions. Among species comparisons often report contrasting relationships between social complexity and brain size, potentially due to confounding effects of phylogeny, morphology and ecology. Here, we explore this relationship in a single fish species, combining behavioural observations and brain measurements of two wild populations of the cichlid *Neolamprologus brevis*, which occupies similar ecological niches across its range but inhabits contrasting social environments depending on local shelter abundance. We quantified social behaviour and brain size to assess whether increased social interactions are associated with greater relative brain size or region-specific neuroanatomical adaptations. We found that individuals from the more socially complex population, which exhibited more frequent social interactions, had significantly larger total brain volumes compared to the less social population. We also found that the more social population exhibited relatively larger telencephalon and smaller hypothalamus volumes, suggesting mosaic adaptation to social demands. Feeding behaviour did not differ between populations, suggesting that differences in energy intake are unlikely to account for brain size variation. By integrating behavioural and neuroanatomical data, our study provides empirical support for the SBH in a natural, within-species comparison.

## 1. Introduction

Vertebrate species exhibit striking variation in brain size, ranging from a few milligrams in small-bodied fish to over 10 kilograms in large mammals. Importantly, larger relative brain size—or the enlargement of specific brain regions—is often associated with enhanced cognitive performance [1–5]. The brain is among the most metabolically costly organs to develop and maintain [6,7], and its size is thought to reflect a trade-off between the cognitive benefits it provides and the energetic costs it incurs. This trade-off may also shape investment in specific brain regions based on their functional relevance, and as such, the relative size of a given brain structure can serve as a proxy for its importance in mediating behavioural responses to environmental and social challenges [8–10].

Several hypotheses have been proposed to explain the evolution of brain size and structure across animal taxa [11–14]. The social brain hypothesis (SBH) proposes that species living in complex social environments evolve larger brains, or enlarged brain regions associated with cognitive processing and decision-making, to meet the increased cognitive demands of social interactions [11]. While many studies support this theory—showing a correlation between brain size and social group size in primates [11], for example—the hypothesis remains contentious. Studies on mammalian carnivores [13] and birds [14] have yielded conflicting results, with some suggesting that ecological and developmental factors may overshadow social influences on brain size. Although large-scale phylogenetic comparisons between species are crucial for revealing genetic adaptations on evolutionary time scales, comparisons within a species between divergent populations can be equally informative and even critically reduce confounding factors such as genomic and morphological variation.

While the SBH was originally developed to explain brain evolution among primates at an interspecific level, the application of this hypothesis has extended to multiple taxa and intraspecific variation. Fish can be a particularly useful group in such studies due to their high brain plasticity [15,16], allowing researchers to investigate the effects of social and ecological factors on neuroanatomy over shorter time scales compared to mammals [17]. Recent studies have highlighted the influence of social environments on fish brain size, with evidence suggesting that higher population densities can lead to increased forebrain sizes and enhanced cognitive abilities [18,19]. However, due to the difficulty in measuring direct social interactions in the wild, most studies have relied on proxies for social complexity, such as group size [4,11,20,21], population density [9,18,19] and mating systems [22–24], rather than direct behavioural measurements. Even fewer have linked observed social behaviours to neuroanatomical variations under natural conditions, leaving the connection between social interactions and brain morphology largely unexplored.

To address this gap, we investigate two divergent populations of the Lake Tanganyika shell-dwelling cichlid *Neolamprologus brevis* that differ markedly in social complexity, one population living in areas of dense shells, allowing multiple conspecifics to live in close proximity (hereafter referred to as the +*social* population), while the other population lives on sparsely populated sand and shell environments, with very few social partners (hereafter referred to as the *−social* population). The pronounced differences in social structure between these populations provide a valuable opportunity to test the SBH at the intraspecific level. By measuring *in situ* behaviour and examining overall brain size and structural variation, we tested whether greater social complexity is linked to either increased total brain volume and/or to a mosaic pattern [25] of brain evolution, characterized by the selective enlargement of functionally critical regions. We hypothesized that the +*social* population would exhibit more frequent social interactions due to the higher density and diversity of neighbouring individuals and would have larger total brain volumes (relative to body size) than individuals from the *−social* population. Additionally, we predicted a relative enlargement of specific brain regions involved in social information processing (such as the telencephalon, which is associated with social decision-making in fish [23,26–29]) in the +*social* population compared with the *−social* population.

## 2. Material and methods

### (a) Study species and ecology

**Figure 1.**
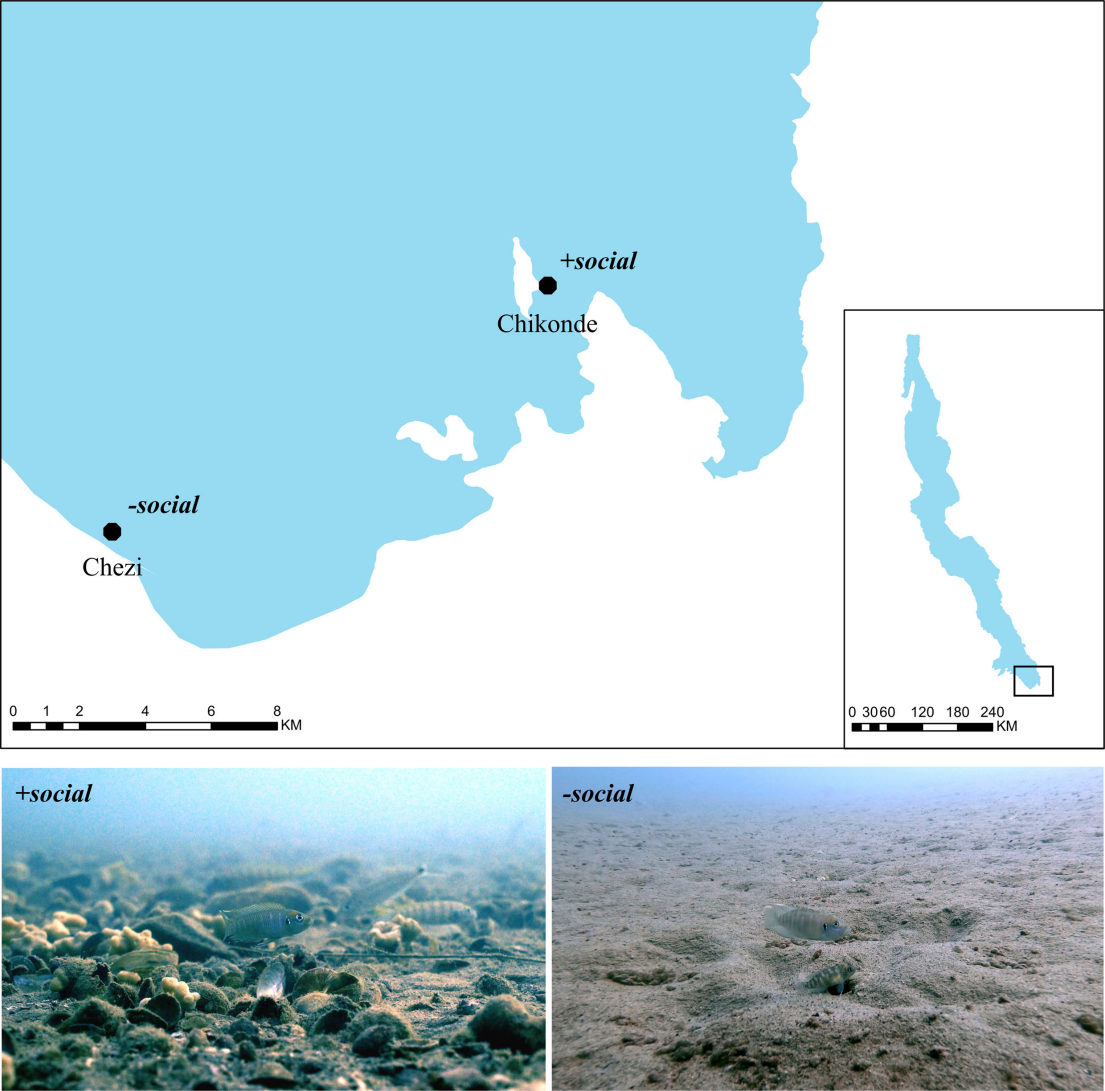
Field sites and two study populations of *Neolamprologus brevis*, illustrating contrasting social environments. Top: map showing the locations of the two study populations at the southern tip of Lake Tanganyika (black dots). Bottom left: representative photo of a +*social* population individual inhabiting continuous shell beds, surrounded by many neighbours, illustrating an environment with higher social complexity (photo credit: Zoë Goverts). Bottom right: representative photo of a −*social* population pair inhabiting sparsely distributed shells on an open sandy substrate, illustrating an environment with lower social complexity (photo credit: Etienne Lein).

We studied two populations of the shell-dwelling cichlid *N. brevis* from Lake Tanganyika (figure 1) that share broadly similar ecological characteristics related to feeding and predation. We selected these geographically distant (14 km) populations to minimize the likelihood of gene flow between them, thereby ensuring that our comparisons reflect population-level differences. Like many other shell-dwelling cichlids, *N. brevis* exhibits extremely restricted home ranges, with daily activities almost entirely confined within a small area (~50 cm radius) around their shells [30,31]. Within this restricted range, individuals primarily feed on plankton from the water column and small benthic invertebrates from substrate [31,32]. Shells also offer essential protection against their main predator, *Lepidiolamprologus elongatus*, a widespread piscivore [31].

Due to differences in shell abundance and spatial distribution, these two populations differ markedly in their social structure (figure 1). In the +*social* population, located near Chikonde village on Mutondwe Island (8°42′43″ S, 31°05′33″ E), individuals inhabit densely packed shell beds (figure 1) where adult males and females are similar in size, preventing cohabitation in a single shell. This lack of obvious sexual dimorphism also makes field-based sex identification challenging. Although individuals do not form stable social groups, the high local density leads to frequent interactions with immediate neighbours, something that we quantify directly in this study. In contrast, the *−social* population, located near Chezi village (8°46′44.9″ S, 31°00′25.2″ E), inhabits a sandy-bottom habitat with sparsely distributed shells (figure 1), typically spaced around more than 10 metres apart. Adult females are smaller than males, and both sexes can cohabit a single ‘home shell’ as breeding pairs.

### (b) Behavioural observation and neighbour density

We quantified social complexity using two complementary metrics: the frequency of direct social interactions and the neighbour density. Data were collected by SCUBA-based *in situ* underwater video recordings (GoPro Hero 6) on unmanipulated *N. brevis* individuals. Focal fish were haphazardly selected, but we attempted to capture a wide size range of adult individuals living in either population. In the +*social* population, nine widely spaced individuals were recorded in May 2022. In the *−social* population, eight individuals were recorded in May 2023. Seven of these were part of distinct breeding pairs, but only one individual per pair was selected for focal observation. The remaining individual was solitary. All focal individuals were located at least 10 metres apart to ensure behavioural independence.

Behaviour recording was conducted using GoPro cameras (Hero 6; 1080 p, 60 fps, wide field of view) placed in front of each fish’s shell and territory. Video was recorded between 10.00 and 11.00 to standardize diurnal activity patterns. Due to field constraints, recording durations varied between 27 and 65 min. To ensure consistency and avoid disturbance artefacts, the first 10 min and last 2 min of each recording were excluded, resulting in scoring windows ranging from 15 to 53 min per fish. Behaviours were scored according to an ethogram adapted from Reyes-Contreras *et al.* [33] and categorized into social and feeding behaviours. Social behaviours included aggression (e.g. ramming, biting, mouth fighting, frontal approaches, S-bend displays), submissive displays (e.g. tail quivering, zig-zag swimming) and affiliative interactions (e.g. joining, bumping, following). Although heterospecific interactions are an important component of social environments in many species [34], interactions between *N. brevis* and other species were exceptionally rare in our observations (*+social* population: 0.025 per min; *−social* population: 0 per min), and thus our quantification of social complexity focused exclusively on conspecific interactions. Feeding behaviours included substrate and plankton pecking, used as a proxy for energy intake. Following each recording, a 50 cm radius ring (area ≈ 0.785 m²) was placed around the focal fish, and the number of conspecific individuals within this area was recorded as the measure of neighbour density.

### (c) Sampling and brain morphology measurements

Brain sampling was conducted on a different set of individuals from those used in behavioural recordings, but all individuals were collected from the same populations and time periods to ensure comparability. In total, 19 individuals from the +*social* population and 24 individuals from the *−social* population were captured and euthanized using an overdose of MS-222 (tricaine methanesulfonate, 1 g l^−1^). Sex was confirmed via gonadal examination post-dissection. Standard length and body weight was measured to the nearest 0.01 mm and 0.001 g, respectively. Fish heads were fixed in a solution of 4% paraformaldehyde and 1% glutaraldehyde in phosphate buffer at 4°C for 24 h. Brains were then extracted and preserved in fresh fixative for 1 week before morphological measurements. Brains were photographed from dorsal, ventral and lateral perspectives using a stereo zoom microscope (Axio Zoom.V16, Zeiss) equipped with a digital camera (Axiocam 503 mono, Zeiss). Five brain regions—the telencephalon, optic tectum, cerebellum, dorsal medulla and hypothalamus—were selected to measure the brain morphology [23]. Each region’s length (L), width (W) and height (H) were measured using ImageJ software [35]. Volumes were calculated using the ellipsoid formula: V = (L × W × H) π/6. Total brain volume was calculated as the sum of the volumes of the five measured brain regions.

### (d) Data analysis

All statistical analyses were conducted in R (v 4.4.1). Brain volumes and body size data were all log-transformed to meet parametric assumptions. Model fits were assessed via the DHARMa package [36]. Descriptive statistics are presented as mean and standard deviation (s.d.). Statistical significance was set at *p* < 0.05.

#### (i) Neighbour density and behaviour frequencies

Neighbour density (count of conspecifics within a 50 cm radius of each focal fish) was compared between the two populations using a generalized linear model (GLM) with a Poisson distribution and log link function using the *glmmTMB* package [37]. To assess differences in the frequencies of feeding and social behaviour between the two populations (*+social* and *−social*, with *−social* as the reference level), separate GLMs were fitted. For feeding behaviour, we fitted a negative binomial model to handle overdispersion, including the count of behaviour as the response variable. We also included population as a categorical predictor as well as the count of neighbours as an additional predictor variable. To account for differences in the time windows when behaviour was scored, we also included observation duration as a model offset (log-transformed). Social behaviour was modelled similarly, with an additional zero-inflation term to handle excess zeros. Sex of the fish could not be determined from the videos, and so sex was not included as a factor in either model.

#### (ii) Total brain volume

We analysed total brain volume using a linear model that controls for allometric scaling by including body size as a covariate [38–40]. We chose body weight over standard length because our weight measurements are more precise than the length measurements, especially given the relatively small sizes of these cichlids; nevertheless, parallel analyses using standard length (electronic supplementary material, table S2 and figure S1) are presented in the electronic supplementary materials, where we also discuss our rationale in greater detail. Body weight was first log-transformed and then meancentred based on the global *N. brevis* body size in our dataset, such that a body weight of 0 refers to an average-sized individual. The model of log-transformed total brain volume included the following predictors: mean-centred log-transformed body weight, social population (*+social* versus *–social*) and sex (male versus female). Inclusion of all pairwise interactions was tested with a likelihood ratio test to check whether each one significantly improved model fit (using the *drop1* function in R), which retained the interaction between body weight and population. Estimated marginal means (EMMs) were calculated using the *emmeans* package [41] for pairwise population comparisons at the average fish size.

#### (iii) Relative brain region volumes

We conducted separate linear models to analyse five brain regions. In each model, the dependent variable was the log-transformed volume of a given brain region, predicted by population and sex and the log-transformed volume of the remaining brain (total brain volume minus the region of interest). Remaining brain volume was used as covariate following prior studies [42,43], to capture brain structure variation and identify potential selective enlargement in functionally relevant regions [44]. This scaling approach tests the mosaic brain hypothesis, which posits that specific brain regions may expand independently while the remainder of the brain remains unchanged [25].

## 3. Results

### (a) Neighbour density and behavioural differences

**Figure 2.**
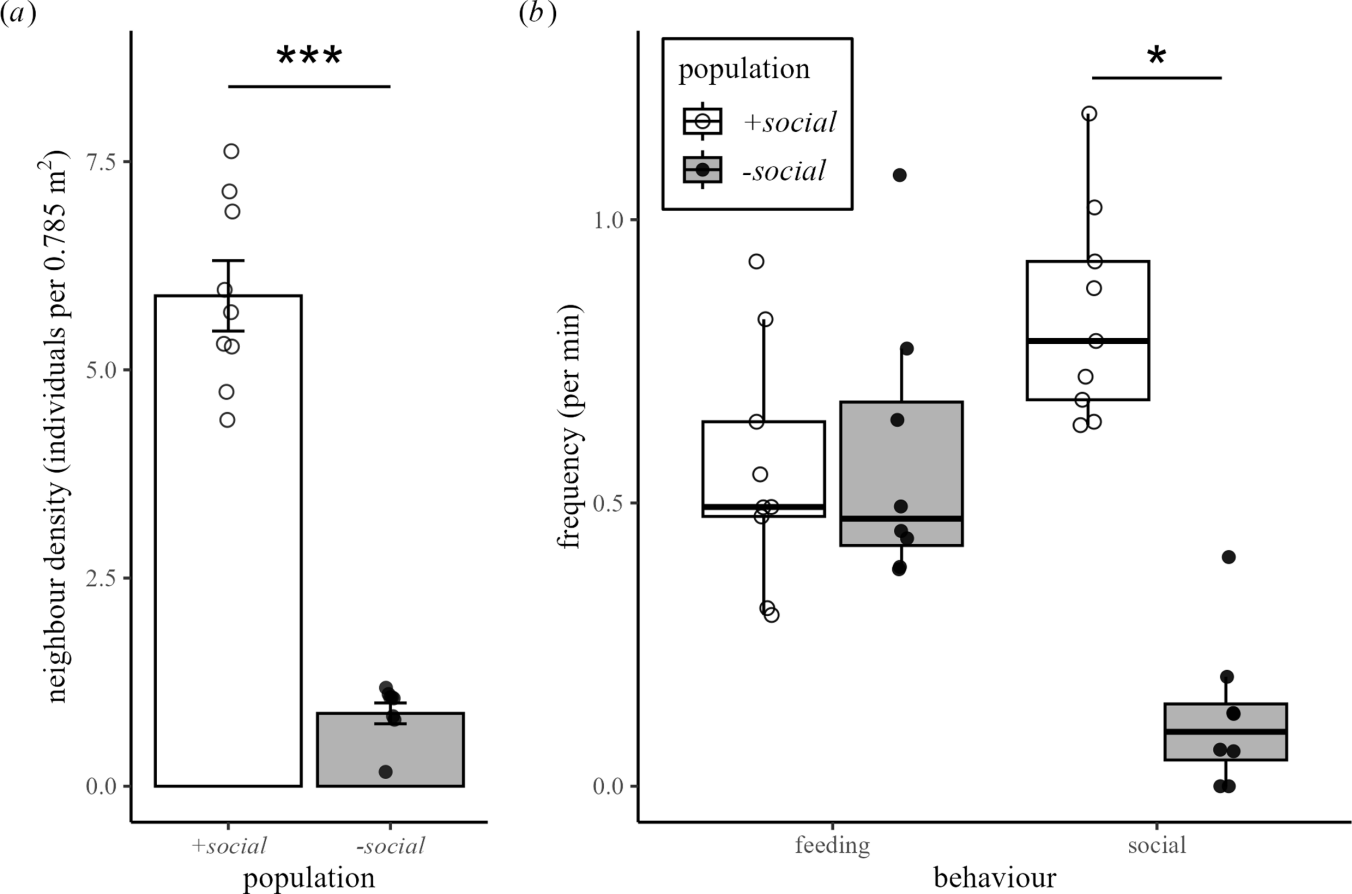
Differences in neighbour densities (the number of neighbours within a 50 cm radius of the focal individual) and behavioural frequencies between +*social* (*n* = 9) and −*social* (*n* = 8) population. (*a*) The +*social* population have more neighbours than the −*social* population (GLM: *** *p* < 0.001). Bar plot showing mean ± s.e. of neighbour density. (*b*) Feeding frequency did not differ between two populations (GLM: *p* = 0.16), whereas the +*social* population exhibited significantly higher frequencies of social behaviour than the −*social* population (GLM: *p < 0.05). Box plots display medians (horizontal line), interquartile ranges (boxes) and data no greater than 1.5 times the interquartile range (whiskers).

We found that neighbour density (number of conspecifics within 50 cm radius of focal individual) was significantly higher in the +*social population* compared to the *−social population* (*+social* versus *−social*: 5.89 ± 1.23 versus 0.88 ± 0.34; GLM: *n* = 17, estimate ± s.e. = 1.91 ± 0.40, z = 4.74, *p* < 0.001; figure 2*a*). Feeding behaviour frequency did not differ significantly between the two populations (*+social* versus *−social*: 0.56 ± 0.21 versus 0.58 ± 0.19 feeding behaviours per minute; GLM: *n* = 17; estimate ± s.e. = −0.68 ± 0.48, z = −1.41, *p* = 0.16; figure 2*b*). Social behaviour was significantly more frequent in the +*social population* compared to the *−social population* (*+social* versus *−social*: 0.83 ± 0.19 versus 0.12 ± 0.13 social behaviours per minute; GLM: *n* = 17; estimate ± s.e. = 2.40 ± 0.38, z = 6.39, *p* < 0.001; figure 2*b*).

### (b) Differences in total brain volume

**Figure 3.**
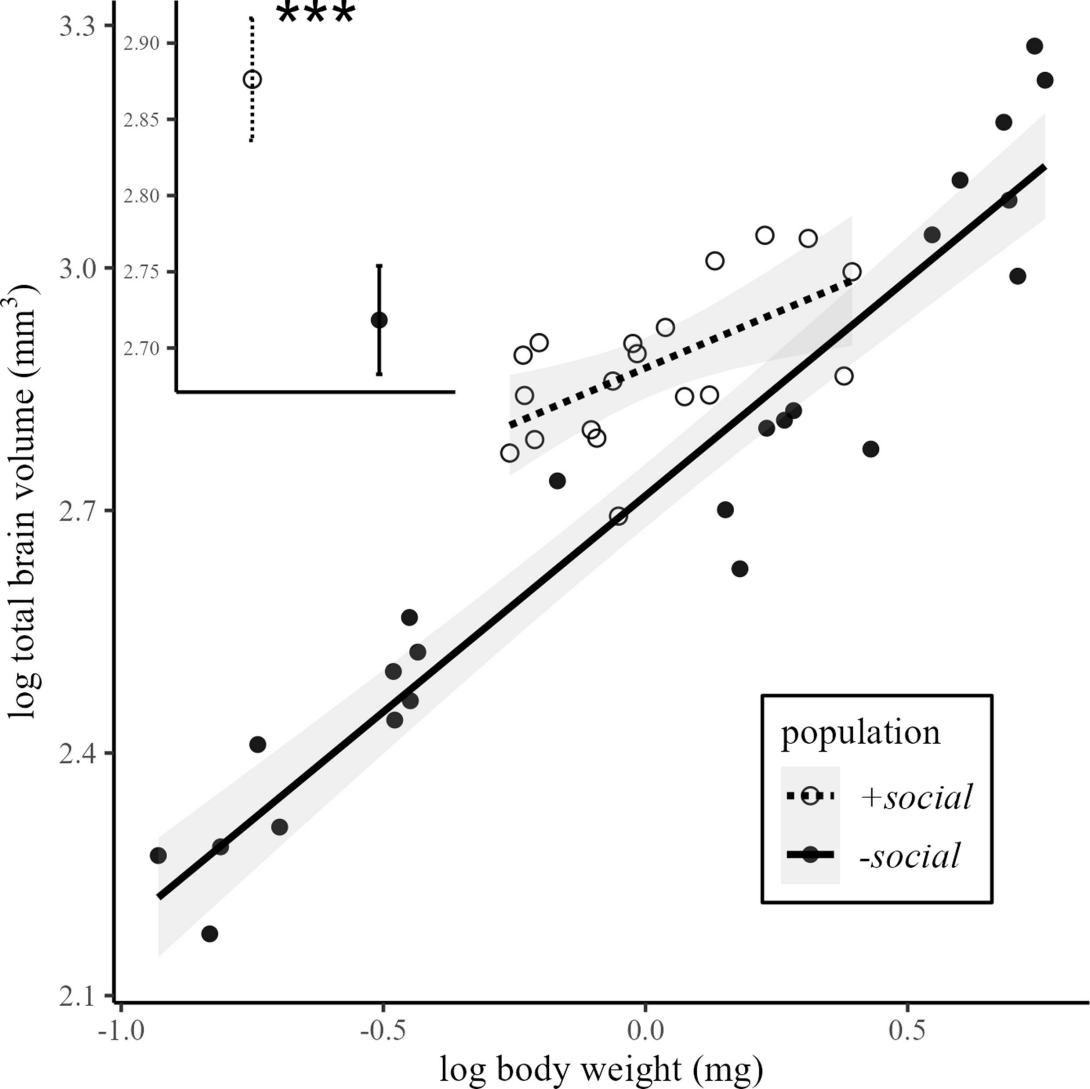
Differences in total brain volume between +*social* (*n* = 19) and −*social* (*n* = 24) populations. The relationship between log-transformed total brain volume and log-transformed body weight between populations. Regression lines represent model predictions with 95% CIs (shaded areas). The inset depicts estimated marginal means (EMMs) of total brain volume at average body weight with 95% CI calculated from the final model, indicating that the +*social* population had significantly larger brains than the −*social* population (post hoc pairwise comparisons: *** *p* < 0.001).

We detected a significant interaction between population and log-transformed body weight on total brain volume (LM, interaction effect: estimate ± s.e. = −0.26 ± 0.10, *t* = 2.44, *n* = 43, *p* < 0.05; figure 3). To assess brain volume differences at a common body size, we calculated EMMs of total brain volume at the average *N. brevis* body weight across the two populations. We found that the +*social* population exhibited significantly larger brain volumes than the *−social* population (post hoc pairwise comparisons: *n* = 43; EMM difference = 0.16 ± 0.03, *p* < 0.001; figure 3, inset). This corresponds to an approximately 17.1% increase in brain volume in the +*social* population for an average-sized *N. brevis* compared with the *−social* population. Sex did not have a significant main effect on brain volume (LM: *n* = 43, estimate ± s.e. = 0.01 ± 0.04, *t* = 0.29, *p* = 0.774; see electronic supplementary material, table S2 for full model statistics and figure S2 for the sex distribution plot).

### (c) Differences in relative brain region volumes

**Figure 4.**
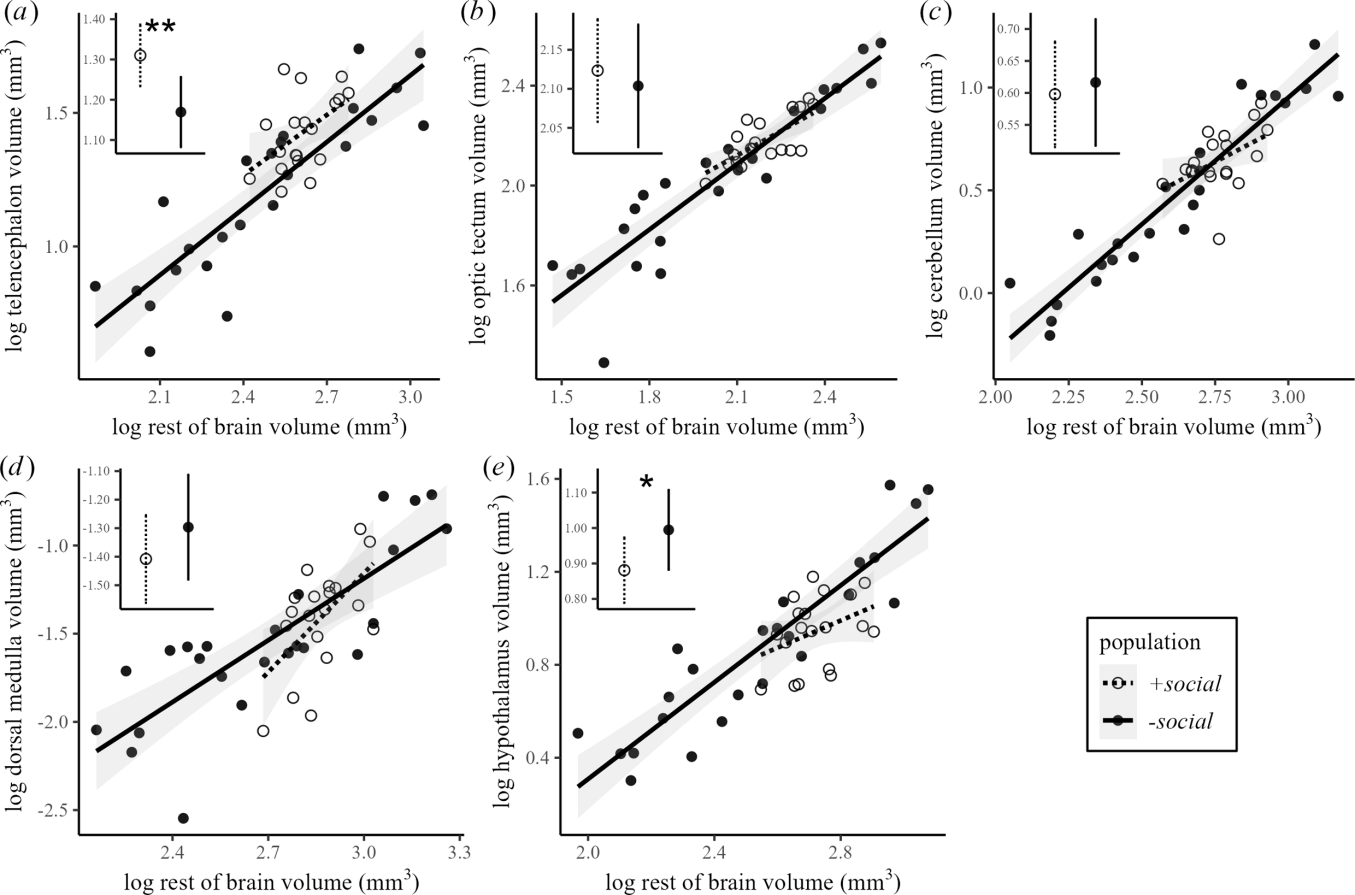
Individual brain region volumes relative to the rest of brain between +*social* (*n* = 19) and –*social* (*n* = 24) populations. Regression lines and 95% CI of log-transformed volume (mm^3^) of the brain region of interest ((*a*) telencephalon, (*b*) optic tectum, (*c*) cerebellum, (*d*) dorsal medulla and (*e*) hypothalamus) on the log-transformed rest of the brain without the volume of the corresponding region (mm^3^). Insets within each plot are the estimated marginal means (EMMs) of each brain region (log) at the mean rest of brain volume (log) for the +*social* and −*social* populations, with 95% CI calculated from the statistical models. The +*social* population exhibited significantly larger telencephalon and smaller hypothalamus volumes relative to the rest of brain compared with the −*social* population (LM: * *p* < 0.05, ** *p* < 0.01; electronic supplementary material, table S2).

The telencephalon was significantly larger in the +*social* population compared with the *−social* population (LM: *n* = 43; estimate ± s.e. = 0.14 ± 0.04, *t* = 3.35, *p* < 0.05; figure 4*a*). In contrast, the hypothalamus was significantly smaller in the +*social* population compared to the *−social* population (LM: *n* = 43; estimate ± s.e. = −0.11 ± 0.05, *t* = −2.13, *p* < 0.05; figure 4*e*). No significant population differences were found in the optic tectum (LM: *n* = 43; estimate ± s.e. = 0.02 ± 0.04, *t* = 0.54, *p* = 0.60), cerebellum (LM: *n* = 43; estimate ± s.e. = −0.02 ± 0.05, *t* = −0.4, *p* = 0.70), or dorsal medulla (LM: *n* = 43; estimate ± s.e. = −0.11 ± 0.09, *t* = −1.29, *p* = 0.21). See electronic supplementary material, table S2 for full model statistics.

## 4. Discussion

In this study, we tested the SBH at the intraspecific level by leveraging natural variation in the frequency of social behaviour among populations of the Lake Tanganyika cichlid fish *N. brevis*. We found that individuals from the +*social* population at Chikonde, which inhabit densely packed shell beds and experience a higher density of conspecific neighbours (averagely 5.89 ± 1.23 in 50 cm radius), exhibited more frequent social interactions (6.7 times more frequent) and larger brain volumes (17.1%) relative to body size compared to individuals from the *−social* population at Chezi, which occupy more sparsely distributed shells on open sandy substrates with few neighbouring individuals (their only neighbours within 50 cm were their breeding partners with whom they shared a shelter). These findings align with the predictions of the SBH, suggesting that increased social complexity is associated with enlarged brain size. Furthermore, individuals from the +*social* population had a relatively larger telencephalon and a smaller hypothalamus compared to those from the *−social* population, suggesting that changes social environment influence both overall brain size and region-specific adaptations may be due to enhanced cognitive demands in complex social environments.

Traditionally, tests of the SBH have assessed social complexity using indirect proxies such as group size [45], population density [18] or mating system (e.g. monogamous versus polygamous [23]). Our study differs from the previous in that we quantified not only the neighbour density but also the frequency of social behaviours with these different social partners, providing a more direct and ecologically relevant assessment of social complexity. We suggest that *N. brevis* in the +*social* population are exposed to a more socially stimulating environment, where individuals encounter a greater number of conspecific neighbours, and interact with them more frequently (due to the populations higher local density and spatial proximity of shell shelters). Maintaining distinct social relationships with many neighbours is thought to be energetically and cognitively costly [46], and brain tissue is metabolically expensive to maintain, imposing strong selective pressures against nonadaptive changes [6,7,47]. Our study species is a continual forager from the benthos and water column, and the similar frequency of feeding behaviour, as well as shared foraging niche among populations suggests energy intake is similar across populations. Differences in brain size may therefore be more likely attributable to increased demands associated with frequent social interactions.

This interpretation—that differences in brain size reflect increased demands associated with social interaction—is further supported by the observed divergence in brain structure. Specifically, individuals from the +*social* population exhibited a larger telencephalon but a smaller hypothalamus compared to those from the *−social* population. Both these regions have been implicated in social behaviour [23]; the telencephalon is involved in mediating complex social behaviours such as decision-making [28,48,49], social recognition [50] and social hierarchy [51], while the hypothalamus regulates endocrine pathways that underlie affiliative behaviours, particularly in reproductive contexts [52–55]. In our study, the +*social* population inhabited socially dense environments where individuals frequently encountered multiple conspecifics, potentially necessitating enhanced cognitive processing related to individual recognition, perception of social status and social decision-making. The *−social* population, on the other hand, consisted mostly of stable mating pairs, which may require greater demands for hormonal regulation associated with affiliative behaviour and reproduction rather than complex social cognition. These findings support a functional trade-off in neural allocation between brain regions, consistent with the mosaic brain hypothesis [25], which posits that brain regions can evolve semi-independently in response to specific ecological or social challenges, often under constraints of total energetic investment.

It is important to note that increases in brain size or specific brain regions may not be driven solely by social complexity but also by environmental or ecological factors, such as habitat complexity or predation pressure [23,56–59]. In this study, we compared two populations without geographic replication, meaning unmeasured environmental factors could have influenced our results. The clever foraging hypothesis, for example, posits that structurally complex habitats promote the evolution of larger brains—particularly in the telencephalon, which is associated with spatial memory and navigation [60–62]. In natural systems, social and physical environmental complexity often covary [63,64], raising the possibility that the observed brain differences in this study could be driven in-part by variation in habitat structure. However, the structural similarity among the environments in our study, both of which consist of snail shells on sandy substrates without large rocks reduces the likelihood of topography playing a major role (e.g. [65]). Moreover, individuals of this species exhibit highly localized movement, typically remaining within a 50 cm radius of their home shell, further reducing the demands on spatial cognition or visual perception. Another potential effect between these two populations is the local predation regime. Predation is known to affect brain size and morphology in fishes, over both evolutionary and developmental time scales [66–70], as well as affecting social organization in Tanganyikan cichlids [71]. While observing direct predation is difficult—and in this study we did not observe any such events—future work would benefit from an explicit analysis of (potential) predation pressure. Specifically, the inclusion of multiple independent +*social* and *–social* populations of *N. brevis* would further elucidate the relationship between social, environmental and ecological effects on brain volume.

A second potential factor to consider is sex, which is known to play a role in the relationship between brain and environment [72]. In our study females of the two populations differ in their size and use of home shells—either sharing them with the male (*−social* population at Chezi), or occupying their own shell (*+social* population at Chikonde). We observed pronounced sexual dimorphism in the *–social* population, but not in the +*social* population. This pattern is likely related to ecological constraints arising from differences in shell availability. In the *−social* population, where shells are scarce, females must remain smaller to allow both the female and male to shelter within the same shell. During sampling, we observed that females were often the first to retreat into the home shell, with males still able to fit inside afterward. In contrast, in the +*social* population, the abundant availability of shells allows every fish to occupy a separate shell, reducing selective pressure for females to remain smaller and resulting in similar body sizes between the sexes. Despite these body size differences, we confirmed that all sampled individuals were sexually mature through gonadal examination, making age or ontogenetic stage unlikely confounding factors. While we did not detect an effect of sex in our analyses, these differences in the use of shells and female body size among the population, and potentially therefore social interactions, warrant further attention in future studies.

Traditionally, the role of sociality in shaping brain size and structure has been tested primarily through interspecific comparisons, which have yielded mixed results. Positive associations between social complexity and brain size have been reported in carnivores and insectivores [73,74], ungulates [75] and primates [76], while similar interspecific comparisons in birds [14] and rodents [77] have either failed to detect such correlations or reported negative associations. These inconsistencies highlight the complex relationship between sociality and brain evolution and underscore the challenges posed by ecological confounding factors—such as foraging niche—inherent in interspecific approaches. Indeed, a recent large-scale comparative study of 1886 bird species found that developmental mode and foraging strategy were stronger predictors of brain size than sociality [14]. This inconsistency is also evident in Lake Tanganyika cichlids. One earlier phylogenetically controlled study of Ectodine species found larger telencephalic size in biparental compared to polygamous species [23], while another analysis of 16 lamprologine species showed that cooperative breeders did not have larger brains than independent breeders [78]. By focusing on naturally occurring variation of social complexity within *N. brevis*, our study was able to avoid many of these interspecific confounds and offers more direct support for the SBH. Although our findings support a positive association between social complexity and brain size, the observational nature of this study precludes any inference of causality. It remains unclear whether increased sociality drives brain enlargement, or whether individuals with larger brains are inherently more adept at navigating complex social environments. Studies tracking neuroanatomical changes in response to experimentally manipulated social environments, or that leverage natural variation within a population, are needed to disentangle these effects.

Overall, our study demonstrates that increased social complexity is associated with larger brain size and specific neuroanatomical adaptations in the cichlid *N. brevis*, providing empirical support for the SBH. Moreover, our findings emphasize the importance of integrating behavioural and neuroanatomical data to validate the social effect on brain anatomy. Finally, our research underscores the value of cichlid fish as a model for studying brain evolution and highlights the potential of using intraspecific design to explore the intricate interplay between sociality and brain morphology.

## Data Availability

All data and R code supporting this study are publicly available in the Dryad Digital Repository [79]. Supplementary material is available online [80].
